# Association of the long fluoroscopy time with factors in contemporary primary percutaneous coronary interventions

**DOI:** 10.1371/journal.pone.0237362

**Published:** 2020-08-10

**Authors:** Satoshi Asada, Kenichi Sakakura, Yousuke Taniguchi, Kei Yamamoto, Takunori Tsukui, Masaru Seguchi, Hiroshi Wada, Shin-ichi Momomura, Hideo Fujita

**Affiliations:** Division of Cardiovascular Medicine, Saitama Medical Center, Jichi Medical University, Shimotsuke, Japan; Baylor Scott and White, Texas A&M College of Medicine, UNITED STATES

## Abstract

**Background:**

Since the long fluoroscopy time in primary PCI for ST-segment elevation myocardial infarction (STEMI) could be an indicator of delayed reperfusion, it should be important to recognize which types of lesions require longer fluoroscopy-time in primary PCI. The purpose of this study was to investigate the association of the long fluoroscopy-time with clinical factors in primary percutaneous coronary interventions (PCI).

**Methods:**

A total of 539 patients who underwent primary PCI were divided into the conventional fluoroscopy-time group (Q1-Q4: n = 434) and the long fluoroscopy-time group (Q5: n = 105) according to the quintile of the total fluoroscopy time in primary PCI. Univariate and multivariate logistic regression analyses were performed to find associations between clinical variables and the long fluoroscopy-time.

**Results:**

In univariate logistic regression analysis, prevalence of diabetes mellitus, hemodialysis, and previous CABG were significantly associated with the long fluoroscopy-time. In addition, complex lesion characteristics such as lesion length, lesion angle, tortuosity, and calcification were associated with the long fluoroscopy-time. In multivariable logistic regression analysis, lesion length [per 10 mm incremental: odds ratio (OR) 1.751, 95% confidence interval (CI) 1.397–2.195, P<0.001], moderate-excessive tortuosity (vs. mild tortuosity: OR 4.006, 95% CI 1.498–10.715, P = 0.006), and moderate to severe calcification (vs. none-mild calcification: OR 1.865, 95% CI 1.107–3.140, P = 0.019) were significantly associated with the long fluoroscopy-time.

**Conclusions:**

In primary PCI for STEMI, diffuse long lesion, tortuosity, and moderate-severe calcification were associated with the long fluoroscopy-time. These complex features require special attention to reduce reperfusion time in primary PCI.

## Introduction

Primary percutaneous coronary intervention (PCI) has dramatically reduced the mortality of ST-segment elevation myocardial infarction (STEMI) [1, 2]. Although most of the culprit lesions for STEMI are totally occluded, it is technically easier to achieve successful reperfusion in primary PCI as compared to PCI to chronic total occlusion (CTO), which are also totally occluded [3, 4]. However, it is sometimes difficult to achieve successful reperfusion even in primary PCI [5]. Delayed reperfusion, which results in poor clinical outcomes [6, 7], can consequently lead to long fluoroscopy-time in primary PCI. Moreover, long fluoroscopy-time would be associated with increased rates of periprocedural complications including early mortality, emergent CABG, contrast-induced nephropathy [6]. Thus, it is important for better patient’s outcomes to recognize which types of lesions require long fluoroscopy-time in primary PCI. The purpose of this study was to investigate the association of the long fluoroscopy-time with clinical factors in primary PCI.

## Methods

### Study lesions

We identified consecutive 624 patients with STEMI in the Saitama Medical Center, Jichi Medical University from January, 2015 to December, 2018. We excluded cases that did not have PCI to the culprit lesion, because of medical therapy alone or surgical therapy to the culprit lesion. We also excluded non-emergent PCI cases, unsuccessful PCI cases, and cases in which ≥2 vessels were treated simultaneously. We defined the long fluoroscopy-time group as the longest quintile of fluoroscopy time (Q5), and defined the conventional fluoroscopy-time group as the other quintiles of fluoroscopy time (Q1-Q4). This study was approved by the Institutional Review Board of Saitama Medical Center, Jichi Medical University, and written informed consent was waived because of the retrospective study design.

### Primary PCI procedures

PCI procedures were performed on a biplane fluoroscopy system (Artis zee BC and Artis zee BA, Siemens, Munich, Germany), with three magnetic fields (16 cm, 20 cm, and 25 cm for Artis zee BC; 16 cm, 22 cm, and 32 cm for Artis zee BA) and a standard image acquisition program at 15 frames per second during cine acquisition and 7.5 frames per second during fluoroscopy. Primary PCI was performed using standard techniques via the radial artery, femoral artery, or rarely brachial artery. First, we advanced a conventional guidewire across the lesion, and used a small (2.0-mm diameter) balloon or thrombus aspiration catheter. The choice of devices was at the discretion of the interventional cardiologist. Activated coagulation time was maintained for >250 seconds during PCI. IVUS was routinely used for almost all lesions.

### Definition

Acute myocardial infarction (AMI) was defined based on universal definition of myocardial infarction [7]. STEMI was diagnosed in the presence of new ST elevation at the J point in at least 2 contiguous leads ≥2 mm (0.2 mV) [8, 9]. Hypertension was defined as medical treatment for hypertension and/or a history of hypertension before admission [10]. Dyslipidemia was defined as a total cholesterol level ≥ 220 mg/dL or low-density lipoprotein cholesterol level ≥ 140 mg/dL or medical treatment for dyslipidemia or a history of dyslipidemia [10]. Diabetes mellitus was defined as a hemoglobin A1c level ≥ 6.5% (as NGSP value) or medical treatment for diabetes mellitus or a history of diabetes mellitus [10]. We calculated the estimated glomerular filtration rate (eGFR) from the serum creatinine level, age, weight, and gender using the following formula: eGFR = 194 × Cr-1.094 × age-0.287 (male), eGFR = 194 × Cr-1.094 × age-0.287 × 0.739 (female) [11].

### Angiographical analysis

Quantitative coronary angiography (QCA) parameters were measured using a cardiovascular angiography analysis system (QAngio XA 7.3, MEDIS Imaging Systems, Leiden, Netherlands). The definition of lesion characteristics including lesion length, eccentricity, tortuosity, lesion angle, ostial lesion, bifurcation lesion has been previously described [12]. In an original ACC/AHA definition, tortuosity was defined as follows: stenoses distal to two bends were defined as moderately tortuous, and those distal to three or more bends were defined as excessive tortuosity [12]. However, since the above definition of tortuosity has poor objectivity, we additionally defined tortuosity as follows: “≤1” moderate to excessive bend (<120°) was defined as mild tortuosity, “2” moderate to excessive bends (<120°) was defined as moderate tortuosity, and “≥3” moderate to excessive bends (<120°) was defined as excessive tortuosity. Calcification was identified as readily apparent radiopacities within the vascular wall at the site of the stenosis, and was classified as none/mild, moderate (radiopacities noted only during the cardiac cycle before contrast injection), and severe (radiopacities noted without cardiac motion before contrast injection generally compromising both sides of the arterial lumen) [13].

### Statistical analysis

Data are shown as the mean ± SD or percentage. Categorical variables are presented as numbers (percentage), and were compared using the Pearson χ2 test. Continuous variables were compared between the groups using the unpaired Student’s t test. Univariate and multivariate logistic regression analyses were performed to find the association between clinical variables and the long fluoroscopy-time. In this model, the long fluoroscopy-time, which was defined as ≥33 minute, was used as the dependent variable. In the multivariate stepwise logistic regression model, the selection of independent variables was derived from the results of univariate logistic regression analysis (P <0.05 in univariate analysis). However, variables with missing values were not included in the model. The multivariate logistic regression analysis with likelihood ratio statistical criteria using backward elimination method was performed. The odds ratio (OR) and the 95% confidence interval (CI) were also calculated. A P value <0.05 was considered statistically significant. We analyzed all data by SPSS ver. 24 for Windows (SPSS, Inc., Chicago, Illinois).

## Results

**Fig 1:**
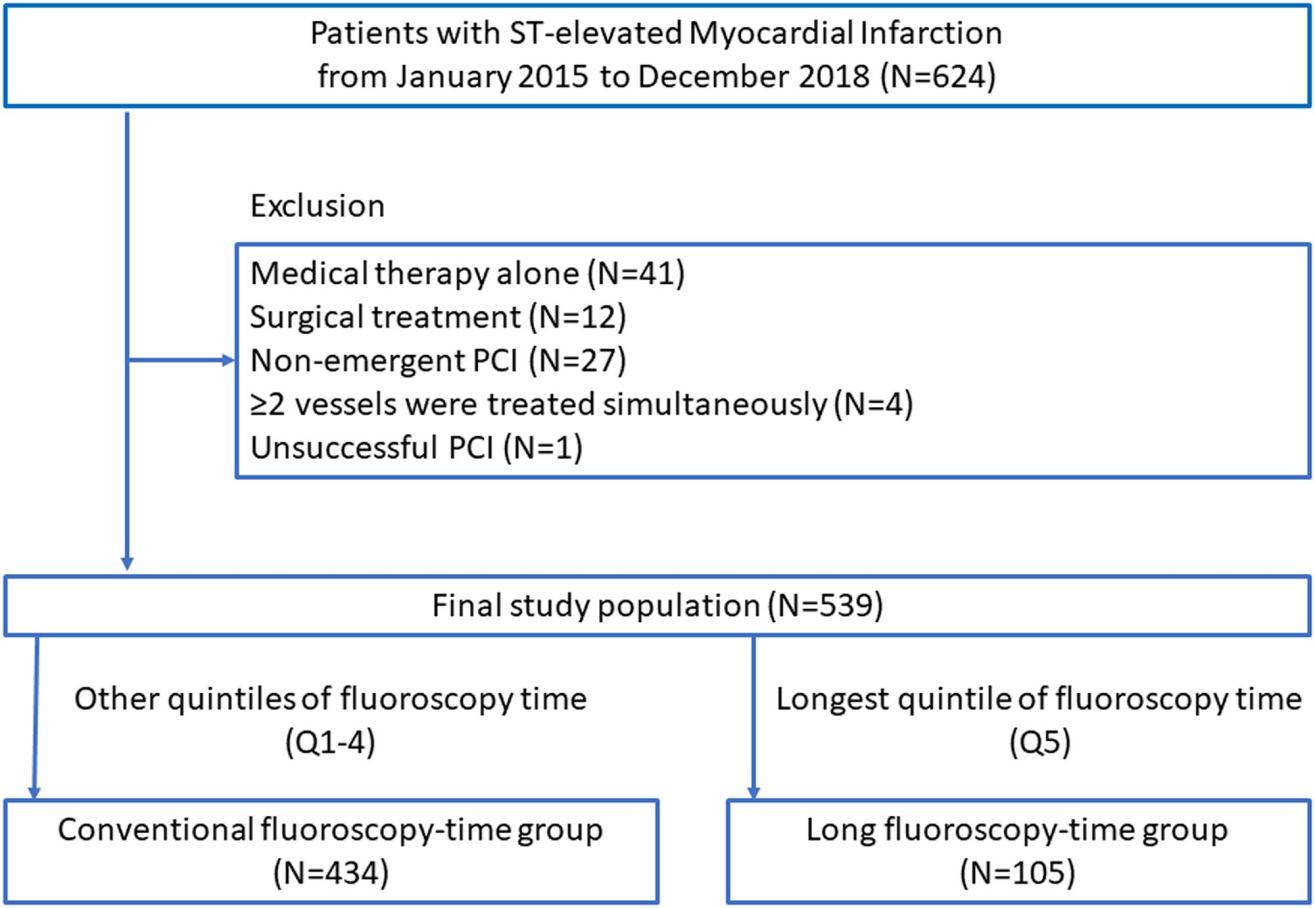
The study flow chart. Abbreviations: CAG = coronary angiography, PCI = percutaneous coronary intervention.

During the study period, we had 624 STEMI cases in our medical center. We excluded 53 cases that did not have PCI to the culprit lesion, 27 non-emergent PCI cases, 4 cases in which ≥2 vessels were treated simultaneously, and 1 unsuccessful PCI case. Our final study population was 539 patients, and divided into the long fluoroscopy-time group (n = 105) and the conventional fluoroscopy-time group (n = 434). The study flowchart is shown in Fig 1.

The comparison of clinical, lesion and procedural characteristics is shown in Table 1. The lesion length was significantly longer in the long fluoroscopy-time group (22.5 ± 13.6 mm) than in the conventional fluoroscopy-time group (15.9 ± 7.2 mm) (P < 0.001). Similarly, there were significant differences in lesion angle, tortuosity, and calcification between the 2 groups.

**Table 1:** Comparison of patient’s characteristics between the conventional fluoroscopy-time group and the long fluoroscopy-time group.

	Conventional fluoroscopy-time group, n = 434	Long fluoroscopy-time group, n = 105	p-value
Age, years	69.2 ± 13.3	68.9 ± 13.6	0.865
Female, n (%)	100 (23.0)	21 (20.0)	0.503
Physical examination			
Height (cm)	162.0 ± 9.7 (n = 428)	163.7 ± 10.4 (n = 104)	0.141
Body weight (kg)	63.1 ± 13.7 (n = 429)	64.9 ± 13.7 (n = 103)	0.234
Systolic blood pressure at admission (mmHg)	136.8 ± 32.2 (n = 418)	138.3 ± 38.5 (n = 97)	0.714
Diastolic blood pressure at admission (mmHg)	80.6 ± 20.1 (n = 411)	82.1 ± 21.1 (n = 96)	0.531
Heart rate at admission (beat per minute)	79.4 ± 21.6 (n = 424)	84.6 ± 25.3 (n = 101)	0.079
Underlying disease			
Hypertension, n (%)	327/423 (77.3)	81 (77.1)	0.972
Diabetes mellitus, n (%)	164/426 (38.5)	54/102(52.9)	0.008
Dyslipidemia, n (%)	233/416 (56.0)	55/103 (53.4)	0.633
Hemodialysis, n (%)	17 (3.9)	10 (9.5)	0.018
History of previous PCI, n (%)	55/433 (12.7)	15/104 (14.4)	0.640
History of previous CABG, n (%)	2 (0.5)	3 (2.9)	0.022
History of previous MI, n (%)	41/433(9.5)	10/104(9.6)	0.963
Current smoker, n (%)	160/422 (37.9)	36/103 (35.0)	0.577
Medication before admission			
Aspirin, n (%)	72/405 (17.8)	19 /96 (19.8)	0.645
Thienopyridine, n (%)	36/404 (8.9)	11/96 (11.5)	0.442
Beta-blocker, n (%)	49/400 (12.3)	19/96 (19.8)	0.054
ACE-inhibitor, ARB, n (%)	132/400 (33)	42/96 (43.8)	0.047
Calcium channel blocker, n (%)	146/401 (36.4)	37/96 (38.5)	0.697
Statin, n (%)	90/401 (22.4)	24/96 (25.0)	0.593
Diuretic, n (%)	35/402 (8.7)	11/96 (11.5)	0.403
Hypoglycemic agents, n (%)	85/402 (21.1)	32/96 (33.3)	0.011
Insulin, n (%)	18/404 (4.5)	7/97 (7.2)	0.262
Laboratory data at admission			
Serum creatinine (mg/dl)	1.1 ± 1.4	1.9 ± 2.6	0.004
Estimated GFR (ml/min/1.73m^2^)	67.3 ± 28.3 (n = 433)	61.7 ± 45.9 (n = 105)	0.009
Hemoglobin (g/dl)	13.6 ± 2.0	13.1 ± 2.1	0.023
BNP (pg/ml)	281 ± 527 (n = 415)	457 ± 641 (n = 95)	0.002
Onset to hospitalization time, n (%)			
0–24 hours	393 (90.6)	85 (81.0)	0.006
Longer than 24 hours	26 (6.0)	9 (8.6)	
Unknown	15 (3.5)	11 (10.5)	
Killip class			
1or2	347 (80.0)	74 (70.5)	0.075
3	32 (7.4)	9 (8.6)	
4	55 (12.7)	22 (21.0)	
Cardiac arrest at prehospital or ER, n (%)	25 (5.8)	15 (14.3)	0.003
Shock vital at prehospital or ER, n (%)	66/427 (15.5)	19/105 (18.1)	0.509
Culprit lesion			
Left main–left anterior descending artery, n (%)	232 (53.5)	52 (49.5)	0.782
Right coronary artery, n (%)	159 (36.6)	42 (40.0)	
Left circumflex, n (%)	41 (9.4)	11 (10.5)	
Graft / other, n (%)	0 (0)	0 (0)	
Not determined, n (%)	2 (0.5)	0 (0)	
Number of narrowed coronary arteries			
1 vessel disease, n (%)	212 (48.8)	40 (38.1)	< 0.001
2 vessel disease, n (%)	147 (33.9)	26 (24.8)	
3 vessel disease, n (%)	75 (17.3)	39 (37.1)	
Left main trunk lesion, n (%)	32 (7.4)	16 (15.2)	0.011
Left main trunk bifurcation lesion, n (%)	17 (3.9)	8 (7.6)	0.106
Ostia lesion, n (%)	21 (4.8)	8 (7.6)	0.257
Bifurcation lesion, n (%)	88 (20.3)	29 (27.6)	0.101
Initial TIMI flow grade of culprit			
0	243 (56.0)	56 (53.3)	0.823
1	44 (10.1)	13 (12.4)	
2	78 (18.0)	17 (16.2)	
3	69 (15.9)	19 (18.1)	
Lesion length (mm)	15.9 ± 7.2	22.5 ± 13.6	< 0.001
Reference diameter (mm)	2.7 ± 0.7	2.6 ± 0.8	0.194
Lesion angle			
Mild (150°<)	192 (44.2)	33 (31.4)	0.003
Moderate(120°<angle≤150°), n (%)	201 (46.3)	51 (48.6)	
Severe (≤120°), n (%)	41 (9.4)	21 (20.0)	
Tortuosity			
Mild tortuosity, n (%)	404 (93.1)	93 (88.6)	0.011
Moderate tortuosity, n (%)	19 (4.4)	3 (2.9)	
Excessive tortuosity, n (%)	11 (2.5)	9 (8.6)	
Eccentricity			
Concentric, n (%)	414 (95.4)		0.386
Eccentric, n (%)	20 (4.6)	98 (93.3)	
Calcification			
None-mild, n (%)	352 (81.1)	66 (62.9)	< 0.001
Moderate, n (%)	54 (12.4)	21 (20.0)	
Severe, n (%)	28 (6.5)	18 (17.1)	
Occurrence of slow flow in PCI, n (%)	36 (8.3)	10 (9.5)	0.686
Approach site			
Trans-radial coronary intervention, n (%)	273/428 (63.8)	56/104 (53.8)	0.033
Trans-femoral coronary intervention, n (%)	9/428 (2.1)	0/104 (0)	
Trans-brachial coronary intervention, n (%)	146/428 (34.1)	48/104 (46.2)	
Change of approach site, n (%)	26 (6.0)	19 (18.1)	< 0.001
PCI procedure			
Plain old balloon angioplasty, n (%)	19 (4.4)	10 (9.5)	0.136
Aspiration only, n (%)	4 (0.9)	1 (1.0)	
Drug coating balloon angioplasty, n (%)	7 (1.6)	1 (1.0)	
Bare metal stent, n (%)	16 (3.7)	0	
Drug eluting stent, n (%)	382 (88.0)	93 (88.6)	
POBA and aspiration, n (%)	2 (0.5)	0	
Other, n (%)	4 (0.9)	0	
Size of guiding catheter (Fr)			
6	320 (73.7)	55 (52.4)	< 0.001
7	114 (26.3)	48 (45.7)	
8	0 (0)	2 (1.9)	
Number of guiding catheters			
1	421 (97.0)	81 (77.1)	< 0.001
2	13 (3.0)	21 (20)	
≥3	0 (0.0)	3 (2.9)	
Number of guide wires			
1	275	22	< 0.001
2	142	52	
≥3	17	31	
Number of stents			
0	41 (9.4)	13 (12.4)	< 0.001
1	371 (85.5)	60 (57.1)	
2	22 (5.1)	31 (29.5)	
≥3	0 (0.0)	1 (1.0)	
Number of balloons			
0	22 (5.1)	1 (1.0)	< 0.001
1	227 (52.3)	28 (26.7)	
2	158 (36.4)	46 (43.8)	
≥3	27 (6.2)	30 (28.6)	
Use of guide-extension-catheter, n(%)	15 (3.5)	26 (24.8)	< 0.001
Use of microcatheter, n(%)	77 (17.7)	62 (59.0)	< 0.001
Aspiration, n (%)	106 (24.4)	32 (30.5)	0.202
Rotablator, n (%)	0 (0.0)	1 (1.0)	0.042
IVUS guide, n (%)	428 (98.6)	105 (100.0)	0.226
OCT/OFDI guide, n (%)	1 (0.2)	0 (0.0)	0.622
Temporary pacemaker, n (%)	39 (9.0)	12 (11.4)	0.443
Intra-aortic balloon pumping support, n (%)	38 (8.8)	24 (22.9)	< 0.001
V-A ECMO, n (%)	14 (3.2)	8 (7.6)	0.041
Amount of contrast media (ml)	120 ± 35	171 ± 59	< 0.001

Data are expressed as the mean ± SD or number (percentage). A Student’s t test was used for normally distributed continuous variables, a Mann-Whitney U test was used for abnormally distributed continuous variables, and a chi-square test was used for categorical variables. There are some missing values for several covariates. In that case, we added sample sizes in the format (N = ***).

PCI indicates percutaneous coronary intervention; CABG, coronary artery bypass grafting; MI, myocardial infarction; STEMI, ST elevation myocardial infarction; NSTEMI, Non-ST elevation myocardial infarction; ACE, Angiotensin converting enzyme; ARB, Angiotensin II receptor blocker; GFR, glomerular filtration rate; BNP, brain natriuretic peptide; ER, emergency room; TIMI, Thrombolysis in myocardial infarction; IVUS, intravascular ultrasound; OCT/OFDI, optical coherence tomography/optical frequency domain imaging; V-A ECMO, veno-arterial extra-corporeal membranous oxygenation.

The univariate logistic regression models to find the factors associated with the long fluoroscopy-time are shown in Table 2. The prevalence of diabetes mellitus, hemodialysis, and previous CABG was significantly associated with the long fluoroscopy-time. In addition, complex lesion characteristics such as lesion length, lesion angle, tortuosity, and calcification were associated with the long fluoroscopy-time. Furthermore, some procedural characteristics such as size of guiding catheter, use of intra-aortic balloon pumping, and use of veno-arterial extra-corporeal membranous oxygenation were also associated with the long fluoroscopy-time.

**Table 2:** Univariate logistic regression analysis to find association with the long fluoroscopy time (≥33 minutes).

Dependent variable: long fluoroscopy time (≥33 minutes)			
Independent variables	Odds ratio	95% confidence interval	P value
***Patient characteristics***			
Age	0.998	0.982–1.014	0.835
Female sex	1.198	0.706–2.030	0.503
Underlying disease			
Height (per 10cm incremental)	1.156	0.938–1.424	0.174
Body weight (per 10kg incremental)	1.112	0.954–1.296	0.173
Systolic blood pressure (mmHg) (per 10mmHg incremental)	1.015	0.951–1.084	0.650
Diastolic blood pressure (mmHg) (per 10mmHg incremental)	1.038	0.932–1.157	0.494
Heart rate (beat per minute) (per 10bpm incremental)	1.103	1.003–1.213	0.043
Underlying disease			
Hypertension	0.991	0.595–1.649	0.972
Diabetes mellitus	1.797	1.163–2.777	0.008
Dyslipidemia	0.900	0.584–1.387	0.633
Hemodialysis	2.582	1.146–5.817	0.022
History of previous MI	1.017	0.492–2.104	0.963
History of previous PCI	1.158	0.626–2.144	0.640
History of previous CABG	6.353	1.048–38.516	0.044
Current smoker	0.880	0.561–1.380	0.577
Medication before admission			
Aspirin	1.141	0.650–2.004	0.646
Thienopyridine	1.323	0.647–2.705	0.443
Beta-blocker	1.768	0.985–3.171	0.056
ACE-inhibitor, ARB	1.579	1.003–2.486	0.049
Calcium channel blocker	1.095	0.692–1.733	0.697
Statin	1.152	0.686–1.934	0.593
Diuretic	1.357	0.662–2.781	0.404
Hypoglycemic agents	1.865	1.146–3.035	0.012
Insulin	1.668	0.676–4.113	0.267
Laboratory data at admission			
Serum creatinine (mg/dl)	1.212	1.095–1.342	<0.001
Estimated GFR (ml/min/1.73m^2^)	0.994	0.987–1.001	0.108
Hemoglobin (g/dl)	0.893	0.806–0.989	0.029
BNP (pg/ml) (per 100pg/ml incremental)	1.047	1.011–1.083	0.009
Onset to hospitalization time			
Longer than 24hours vs. 0–24 hours	0.625	0.283–1.381	0.245
Killip class			
3–4 vs. 1–2	1.827	1.055–3.161	0.031
Cardiac arrest at prehospital or ER	2.727	1.382–5.380	0.004
Shock vital at prehospital or ER	1.208	0.689–2.120	0.509
***Lesion characteristics***			
Culprit lesion			
Left main—left anterior descending artery vs. Others	0.854	0.558–1.309	0.469
Number of narrowed coronary arteries			
3 vessel disease vs. 1 or 2 vessel disease	2.828	1.772–4.515	<0.001
Left main trunk lesion	2.258	1.188–4.294	0.013
Left main trunk bifurcation lesion	2.023	0.849–4.823	0.112
Ostia lesion	1.622	0.698–3.771	0.261
Bifurcation lesion	1.500	0.921–2.443	0.103
Initial TIMI flow grade of culprit			
3 vs. 0–2	1.169	0.668–2.045	0.585
Lesion length (mm) (per 10mm incremental)	1.791	1.448–2.216	<0.001
Reference diameter (mm)	0.813	0.595–1.112	0.195
Lesion angle			
Severe (≤120°) vs. Mild-moderate(120°<)	2.396	1.347–4.264	0.003
Tortuosity			
Moderate-excessive tortuosity vs. Mild tortuosity	3.605	1.454–8.942	0.006
Eccentricity			
Eccentric vs. Concentric	1.479	0.608–3.595	0.388
Calcification			
Moderate-severe vs. None-mild	2.537	1.596–4.031	<0.001
Occurrence of slow flow in PCI	1.164	0.558–2.428	0.686
***Procedural characteristics***			
Approach site			
Trans radial coronary intervention vs. TFI or TBI	0.662	0.430–1.021	0.062
Change of approach site	3.467	1.836–6.546	<0.001
PCI procedure			
Drug eluting stent vs. Others	1.055	0.541–2.056	0.875
Size of guiding catheter			
≥7 vs. 6Fr	2.552	1.646–3.957	<0.001
Number of guiding catheter			
≥2 vs. 1	9.595	4.691–19.627	<0.001
Number of wire			
≥2 vs. 1	6.525	3.922–10.855	<0.001
Number of stent			
≥2 vs. 0–1	8.209	4.513–14.916	<0.001
Number of balloon			
≥3 vs. 0–2	6.030	3.392–10.719	<0.001
Use of extension guide catheter	9.193	4.660–18.136	<0.001
Use of micro catheter	6.685	4.219–10.593	<0.001
Use of aspiration catheter	1.356	0.848–2.170	0.203
Temporary pacemaker	1.307	0.659–2.593	0.444
Intra-aortic balloon pumping support	3.088	1.756–5.428	<0.001
V-A ECMO	2.474	1.010–6.063	0.048

The multivariate stepwise logistic regression analysis to find the factors associated with the long fluoroscopy-time is shown in Table 3. The stepwise model included the following variables: previous CABG, serum creatinine levels, hemoglobin levels, Killip class, cardiac arrest at prehospital or emergency room, number of narrowed coronary arteries, left main trunk lesion, lesion length, lesion angle, tortuosity, calcification, and size of guiding catheters. Serum creatinine(OR 1.139, 95% CI 1.016–1.277, P = 0.026), cardiac arrest at prehospital or ER(OR 2.594, 95% CI 1.205–5.584, P = 0.015), triple vessels disease (vs. 1 or 2 vessel diseases: OR 1.772, 95% CI 1.052–2.983, P = 0.031), lesion length (per 10 mm incremental: OR 1.751, 95% CI 1.397–2.195, P < 0.001), moderate-excessive tortuosity (vs. mild tortuosity: OR 4.006, 95% CI 1.498–10.715, P = 0.006), moderate to severe calcification (vs. none-mild calcification: OR 1.865, 95%CI 1.107–3.140, P = 0.019), and size of guiding catheter ≥7 Fr (vs. 6 Fr: OR 1.751, 95% CI 1.064–2.883, P = 0.028) were significantly associated with the long fluoroscopy-time in this final model.

**Table 3:** Multivariate logistic regression analysis model to find association with the long fluoroscopy time (≥33 minutes).

Dependent variable: long fluoroscopy time (≥33 minutes)			
Independent variables	Odds ratio	95% confidence interval	P value
Serum creatinine (mg/dl)	1.139	1.016–1.277	0.026
Cardiac arrest at prehospital or ER	2.594	1.205–5.584	0.015
Triple vessels disease (vs. 1 or 2 vessel diseases)	1.772	1.052–2.983	0.031
Lesion length (mm) (per 10mm incremental)	1.751	1.397–2.195	<0.001
Moderate-excessive tortuosity (vs. mild tortuosity)	4.006	1.498–10.715	0.006
Moderate-severe calcification (vs. none-mild)	1.865	1.107–3.140	0.019
Size of guiding catheter ≥7 Fr (vs. 6 Fr)	1.751	1.064–2.883	0.028

The stepwise model includes the following variables: History of previous CABG, Serum creatinine (mg/dl), Hemoglobin (g/dl), Killip class, Cardiac arrest at prehospital or ER, Number of narrowed coronary arteries, Left main trunk lesion, Lesion length (mm) (per 10mm incremental), Lesion angle, Tortuosity, Calcification, Size of guiding catheter.

## Discussion

The present study included 539 patients who underwent primary PCI for STEMI, which were divided into the conventional fluoroscopy-time group (n = 434) and the long fluoroscopy-time group (n = 105). In the multivariate stepwise logistic regression analysis, serum creatinine, cardiac arrest at prehospital or ER, three vessel disease, diffuse long lesion, moderate to excessive tortuosity, moderate to severe calcification, and big size of guiding catheter were associated with the long fluoroscopy-time, suggesting that complex lesion characteristics required longer fluoroscopy-time in primary PCI.

Although there were several studies to find determinants of the long fluoroscopy time in PCI [14, 15], there were few studies to find that in primary PCI for STEMI. In the present study, lesion length was significantly associated with the long fluoroscopy time. Since the very beginning of PCI, lesion length has been considered to be an important feature reflecting the difficulty of PCI [12, 16]. We previously reported that total fluoroscopy time was clearly stratified according to the ACC/AHA type classification of coronary lesions such as type A, type B, and type C [17], and lesion length has been a main component of the ACC/AHA type classification system. Even in the current PCI, strong back-up support should be necessary to deliver devices to the diffuse long lesion, which sometimes requires guide-extension-catheters, buddy wire technique, and anchoring technique [18]. Furthermore, diffuse long lesion may require an additional stent to cover the whole lesion. Therefore, diffuse long lesion would require longer fluoroscopy time.

In the present study, moderate-excessive tortuosity was also associated with the long fluoroscopy time. Severe vessel tortuosity has been identified as a predictor of unsuccessful PCI [19]. Nikolsky et al. analyzed the reasons of stent deployment failure using 151 cases, and reported that the dominant reason (92%) of stent deployment failure was unsuccessful to deliver the stent to the lesion site, because of severe tortuosity and/or calcification [20]. Ho, et al. reported that the current thin-strut stent had better deliverability to coronary lesions, but required “five-in-six” system to facilitate stent delivery for severely tortuous lesions [21]. Thus, stent delivery to excessive tortuous lesions still requires additional efforts and the additional fluoroscopy time. Our results suggest that excessive tortuosity can be a problem even in contemporary primary PCI.

Similarly, calcification was significantly associated with the long fluoroscopy time in the present study. Since severe calcification was closely associated with difficulty of device delivery [18, 21], it was expected that longer fluoroscopy time was required in severely calcified lesions. Furthermore, severe calcification can be a cause of balloon underexpansion or balloon rupture [22, 23], which may require additional non-compliant balloons, scoring balloons, or debulking devices [24]. Such additional procedures should require longer fluoroscopy time. Since only 1 case (0.2%) required rotablator in our study population, even calcification that did not need rotablator could be a cause of difficulty in primary PCI.

Serum creatinine was associated with the long fluoroscopy-time. Increased serum levels is known to be associated with advanced atherosclerosis [25, 26]. Advance atherosclerosis could be a cause of complex coronary lesions, which might require additional fluoroscopy time. Cardiac arrest at prehospital or ER was also significantly associated with the long fluoroscopy-time. If vital signs were unstable in catheter laboratories, it should be necessary to stabilize severe conditions using vasopressor agents, IABP, or V-A ECMO, which also require some fluoroscopy time. Gupta N et al. showed that patients with cardiac arrest had more complex coronary lesions [27]. Similarly, triple vessel disease was associated with the long fluoroscopy-time. Triple vessel disease indicates the progression of coronary atherosclerosis. Sorajja P et al. reported that extensive disease in vessels remote from the infarct-related artery was associated with unsuccessful reperfusion in primary PCI [28], which would require longer fluoroscopy time. Lastly, larger size of guiding catheter was significantly associated with the long fluoroscopy-time. However, interpretation of this result should be careful. Since larger guiding catheter size has strong back-up force, we selected larger guiding catheter for lesions with more complex disease. Therefore, larger size of guiding catheter would probably be an effect rather than a cause of longer fluoroscopy-time.

Clinical implications of the present study should be noted. Since primary PCI is performed irrespective of on/off-hours [29], junior operators often perform primary PCI with or without senior operators. Although most lesions may be managed by junior operators, some lesions would require experienced senior operators. In fact, Chen, et al. reported that the significant reduction of in-hospital death was observed when primary PCI was performed by trained operators as compared to untrained operators [30]. However, it may be difficult for junior operators to differentiate which lesions are really difficult. The present study may provide practical information which lesions are really difficult for junior operators in primary PCI. If the lesion are diffuse long, severely tortuous, or severely calcified in coronary angiography, junior operators should ask senior operators of backup support, if possible. Otherwise, junior operators should discuss the strategy to such tough lesions in primary PCI with senior operators beforehand.

### Study limitations

First, because this study was a single-center retrospective observational study, there is a risk of patient selection bias. Second, we arbitrary defined the long fluoroscopy-time as the longest quintile of the fluoroscopy time, because there were no established cut-off points for the long fluoroscopy time, which were also used in early literatures [31–33]. Moreover, if we defined the long fluoroscopy-time as the longest quartile or the longest sextile, the main parameters such as lesion length, tortuosity, and calcification remained significant determinants of the long fluoroscopy time (data was only shown in the peer review process). Third, since total occlusion was frequently observed in the culprit lesion of STEMI, QCA parameters were measured after the acquisition of reperfusion (after ballooning or thrombectomy). Therefore, these QCA parameters might not reflect the original occlusion length or reference diameter.

## Conclusions

In primary PCI for STEMI, serum creatinine, cardiac arrest at prehospital or ER, three vessel disease, diffuse long lesion, moderate-excessive tortuosity, severe calcification were associated with the long fluoroscopy-time. These complex features require special attention to reduce reperfusion time in primary PCI.

## Supporting information

S1 File (XLSX)

## References

[1] Keeley EC, Boura JA, Grines CL. Primary angioplasty versus intravenous thrombolytic therapy for acute myocardial infarction: a quantitative review of 23 randomised trials. Lancet (London, England). 2003;361(9351):13–20. Epub 2003/01/09. 10.1016/s0140-6736(03)12113-7 . 12517460

[2] Hochman JS, Sleeper LA, Webb JG, Sanborn TA, White HD, Talley JD, et al Early revascularization in acute myocardial infarction complicated by cardiogenic shock. SHOCK Investigators. Should We Emergently Revascularize Occluded Coronaries for Cardiogenic Shock. The New England journal of medicine. 1999;341(9):625–34. Epub 1999/08/26. 10.1056/NEJM199908263410901 . 10460813

[3] Bauer T, Hochadel M, Brachmann J, Schachinger V, Boekstegers P, Zrenner B, et al Use and outcome of radial versus femoral approach for primary PCI in patients with acute ST elevation myocardial infarction without cardiogenic shock: results from the ALKK PCI registry. Catheterization and cardiovascular interventions: official journal of the Society for Cardiac Angiography & Interventions. 2015;86 Suppl 1:S8–14. Epub 2015/05/07. 10.1002/ccd.25987 . 25945803

[4] Suzuki Y, Tsuchikane E, Katoh O, Muramatsu T, Muto M, Kishi K, et al Outcomes of Percutaneous Coronary Interventions for Chronic Total Occlusion Performed by Highly Experienced Japanese Specialists: The First Report From the Japanese CTO-PCI Expert Registry. JACC Cardiovascular interventions. 2017;10(21):2144–54. Epub 2017/10/23. 10.1016/j.jcin.2017.06.024 . 29055764

[5] Levi A, Kornowski R, Vaduganathan M, Eisen A, Vaknin-Assa H, Abu-Foul S, et al Incidence, predictors, and outcomes of failed primary percutaneous coronary intervention: a 10-year contemporary experience. Coronary artery disease. 2014;25(2):145–51. Epub 2013/11/28. 10.1097/MCA.0000000000000065 . 24281252

[6] Nikolsky E, Pucelikova T, Mehran R, Balter S, Kaufman L, Fahy M, et al An evaluation of fluoroscopy time and correlation with outcomes after percutaneous coronary intervention. The Journal of invasive cardiology. 2007;19(5):208–13. Epub 2007/05/04. . 17476034

[7] Thygesen K, Alpert JS, Jaffe AS, Chaitman BR, Bax JJ, Morrow DA, et al Fourth Universal Definition of Myocardial Infarction (2018). Journal of the American College of Cardiology. 2018;72(18):2231–64. Epub 2018/08/30. 10.1016/j.jacc.2018.08.1038 . 30153967

[8] Alpert JS, Thygesen K, Antman E, Bassand JP. Myocardial infarction redefined—a consensus document of The Joint European Society of Cardiology/American College of Cardiology Committee for the redefinition of myocardial infarction. Journal of the American College of Cardiology. 2000;36(3):959–69. Epub 2000/09/15. 10.1016/s0735-1097(00)00804-4 . 10987628

[9] O'Gara PT, Kushner FG, Ascheim DD, Casey DE Jr, Chung MK, de Lemos JA, et al 2013 ACCF/AHA guideline for the management of ST-elevation myocardial infarction: a report of the American College of Cardiology Foundation/American Heart Association Task Force on Practice Guidelines. Journal of the American College of Cardiology. 2013;61(4):e78–e140. Epub 2012/12/22. 10.1016/j.jacc.2012.11.019 . 23256914

[10] Watanabe Y, Sakakura K, Taniguchi Y, Yamamoto K, Wada H, Momomura SI, et al Determinants of Slow Flow in Percutaneous Coronary Intervention to the Culprit Lesion of Non-ST Elevation Myocardial Infarction. International heart journal. 2018;59(6):1237–45. Epub 2018/10/12. 10.1536/ihj.18-050 . 30305588

[11] Matsuo S, Imai E, Horio M, Yasuda Y, Tomita K, Nitta K, et al Revised equations for estimated GFR from serum creatinine in Japan. American journal of kidney diseases: the official journal of the National Kidney Foundation. 2009;53(6):982–92. Epub 2009/04/03. 10.1053/j.ajkd.2008.12.034 . 19339088

[12] Ellis SG, Vandormael MG, Cowley MJ, DiSciascio G, Deligonul U, Topol EJ, et al Coronary morphologic and clinical determinants of procedural outcome with angioplasty for multivessel coronary disease. Implications for patient selection. Multivessel Angioplasty Prognosis Study Group. Circulation. 1990;82(4):1193–202. Epub 1990/10/01. 10.1161/01.cir.82.4.1193 . 2401060

[13] Mintz GS, Popma JJ, Pichard AD, Kent KM, Satler LF, Chuang YC, et al Patterns of calcification in coronary artery disease. A statistical analysis of intravascular ultrasound and coronary angiography in 1155 lesions. Circulation. 1995;91(7):1959–65. Epub 1995/04/01. 10.1161/01.cir.91.7.1959 . 7895353

[14] Mujtaba SF, Saghir T, Sial JA, Rizvi NH. Procedural determinants of fluoroscopy time in patients undergoing cardiac catheterization. Pakistan journal of medical sciences. 2019;35(1):166–71. Epub 2019/03/19. 10.12669/pjms.35.1.294 . 30881417 PMC6408651

[15] Fazel R, Curtis J, Wang Y, Einstein AJ, Smith-Bindman R, Tsai TT, et al Determinants of fluoroscopy time for invasive coronary angiography and percutaneous coronary intervention: insights from the NCDR((R)). Catheterization and cardiovascular interventions: official journal of the Society for Cardiac Angiography & Interventions. 2013;82(7):1091–105. Epub 2013/05/25. 10.1002/ccd.24996 . 23703793

[16] Ryan TJ, Faxon DP, Gunnar RM, Kennedy JW, King SB 3rd, Loop FD, et al Guidelines for percutaneous transluminal coronary angioplasty. A report of the American College of Cardiology/American Heart Association Task Force on Assessment of Diagnostic and Therapeutic Cardiovascular Procedures (Subcommittee on Percutaneous Transluminal Coronary Angioplasty). Circulation. 1988;78(2):486–502. Epub 1988/08/01. 10.1161/01.cir.78.2.486 . 2969312

[17] Sakakura K, Ako J, Wada H, Kubo N, Momomura S. ACC/AHA classification of coronary lesions reflects medical resource use in current percutaneous coronary interventions. Catheterization and cardiovascular interventions: official journal of the Society for Cardiac Angiography & Interventions. 2012;80(3):370–6. Epub 2011/08/02. 10.1002/ccd.23270 . 21805596

[18] Chawla R, Ahamad W, Sharma V. Techniques to overcome difficulty in device deliverability to lesion in complex PCI. Current cardiology reviews. 2019 Epub 2019/10/28. 10.2174/1573403x15666191018105627 . 31648644 PMC7460703

[19] Gibson CM, Bigelow B, James D, Tepper MR, Murphy SA, Kirtane AJ, et al Association of lesion complexity following fibrinolytic administration with mortality in ST-elevation myocardial infarction. The American journal of cardiology. 2004;94(1):108–11. Epub 2004/06/29. 10.1016/j.amjcard.2004.03.038 . 15219518

[20] Nikolsky E, Gruberg L, Pechersky S, Kapeliovich M, Grenadier E, Amikam S, et al Stent deployment failure: reasons, implications, and short- and long-term outcomes. Catheterization and cardiovascular interventions: official journal of the Society for Cardiac Angiography & Interventions. 2003;59(3):324–8. Epub 2003/06/25. 10.1002/ccd.10543 . 12822150

[21] Ho HH, Jafary FH, Loh KK, Tan JK, Ooi YW, Ong PJ. Deliverability of integrity coronary stents in severely tortuous coronary arteries: a preliminary experience. The Journal of invasive cardiology. 2012;24(12):650–4. Epub 2012/12/12. . 23220980

[22] Kobayashi Y, Okura H, Kume T, Yamada R, Kobayashi Y, Fukuhara K, et al Impact of target lesion coronary calcification on stent expansion. Circulation journal: official journal of the Japanese Circulation Society. 2014;78(9):2209–14. Epub 2014/07/16. 10.1253/circj.cj-14-0108 . 25017740

[23] Simpfendorfer CC, Dimas AP, Zaidi A, Hollman J, Franco I, Knezinek V, et al Balloon rupture during coronary angioplasty. Angiology. 1986;37(11):828–31. Epub 1986/11/01. 10.1177/000331978603701106 . 2947525

[24] Hirai K, Sakakura K, Watanabe Y, Taniguchi Y, Yamamoto K, Wada H, et al Determinants of high device cost in current percutaneous coronary interventions. Cardiovascular revascularization medicine: including molecular interventions. 2018;19(5 Pt B):607–12. Epub 2018/01/24. 10.1016/j.carrev.2017.12.007 . 29358042

[25] Keane WF, Kasiske BL, O'Donnell MP. Lipids and progressive glomerulosclerosis. A model analogous to atherosclerosis. American journal of nephrology. 1988;8(4):261–71. Epub 1988/01/01. 10.1159/000167599 . 3055991

[26] Ninomiya T, Kiyohara Y, Tokuda Y, Doi Y, Arima H, Harada A, et al Impact of kidney disease and blood pressure on the development of cardiovascular disease: an overview from the Japan Arteriosclerosis Longitudinal Study. Circulation. 2008;118(25):2694–701. Epub 2008/12/25. 10.1161/CIRCULATIONAHA.108.792903 . 19106392

[27] Gupta N, Kontos MC, Gupta A, Dai D, Vetrovec GW, Roe MT, et al Characteristics and outcomes in patients undergoing percutaneous coronary intervention following cardiac arrest (from the NCDR). The American journal of cardiology. 2014;113(7):1087–92. Epub 2014/02/12. 10.1016/j.amjcard.2013.12.014 . 24513475

[28] Sorajja P, Gersh BJ, Cox DA, McLaughlin MG, Zimetbaum P, Costantini C, et al Impact of multivessel disease on reperfusion success and clinical outcomes in patients undergoing primary percutaneous coronary intervention for acute myocardial infarction. European heart journal. 2007;28(14):1709–16. Epub 2007/06/09. 10.1093/eurheartj/ehm184 . 17556348

[29] Lattuca B, Kerneis M, Saib A, Nguyen LS, Payot L, Barthelemy O, et al On- Versus Off-Hours Presentation and Mortality of ST-Segment Elevation Myocardial Infarction Patients Treated With Primary Percutaneous Coronary Intervention. JACC Cardiovascular interventions. 2019;12(22):2260–8. Epub 2019/11/05. 10.1016/j.jcin.2019.07.017 . 31678083

[30] Chen M, Kan J, Zhang JJ, Tian N, Ye F, Yang S, et al Improvement of clinical outcome in patients with ST-elevation myocardial infarction between 1999 and 2016 in China: The Prospective, Multicentre Registry MOODY study. European journal of clinical investigation. 2020;50(2):e13197 Epub 2019/12/29. 10.1111/eci.13197 . 31883102

[31] Wehinger A, Kastrati A, Elezi S, Baum H, Braun S, Neumann FJ, et al Lipoprotein(a) and coronary thrombosis and restenosis after stent placement. Journal of the American College of Cardiology. 1999;33(4):1005–12. Epub 1999/03/26. 10.1016/s0735-1097(98)00684-6 . 10091828

[32] Groenland CNL, Termorshuizen F, Rietdijk WJR, van den Brule J, Dongelmans DA, de Jonge E, et al Emergency Department to ICU Time Is Associated With Hospital Mortality: A Registry Analysis of 14,788 Patients From Six University Hospitals in The Netherlands. Critical care medicine. 2019;47(11):1564–71. Epub 2019/08/09. 10.1097/CCM.0000000000003957 . 31393321 PMC6798749

[33] Khera AV, Emdin CA, Drake I, Natarajan P, Bick AG, Cook NR, et al Genetic Risk, Adherence to a Healthy Lifestyle, and Coronary Disease. The New England journal of medicine. 2016;375(24):2349–58. Epub 2016/12/14. 10.1056/NEJMoa1605086 . 27959714 PMC5338864

